# A Complicated Case of Strangulated Inguinal Hernia of the Sigmoid Colon With Secondary Ischemic-Compromise of Scrotal Tissue: A Multi-Disciplinary Surgical Approach

**DOI:** 10.7759/cureus.48510

**Published:** 2023-11-08

**Authors:** Muzi Meng, Yonas Teklu, Harsh R Parikh, Gagan Sathya Prakash, Joseph Silletti, Ajit Singh

**Affiliations:** 1 School of Medicine, American University of the Carribean, Cupecoy, SXM; 2 General Surgery, BronxCare Health System, Bronx, USA; 3 Department of Surgery, Regions Hospital, St. Paul, USA; 4 Department of Orthopedic Surgery, University of Minnesota School of Medicine, Minneapolis, USA; 5 General Surgery/Internal Medicine, BronxCare Health System, Bronx, USA; 6 School of Medicine, St. George's University, Grenada, GRD; 7 Urology, BronxCare Health System, Bronx, USA

**Keywords:** sigmoid-end colostomy, orchiectomy, fournier's gangrene (fg), emergency exploratory laparotomy, strangulated inguinal hernia

## Abstract

Inguinal hernia is amongst the most common acute abdominal disease that presents in the Emergency Department (ED). Pathologically, it involves the displacement and herniation of abdominal, pelvic, or groin tissue through weaknesses in the abdominal wall. Many inguinal hernias are simple and asymptomatic, managed conservatively without the need for surgical intervention. However, under rare circumstances, hernias are susceptible to significant complications requiring emergent surgery. This report follows the case of a 61-year-old Hispanic-American male presenting to the ED with signs of a complex strangulated inguinal hernia and consequent infarction of the testis with Fournier’s Gangrene. Clinical evaluation elucidated a one-week worsening abdominal pain, non-reducible painful inguinal hernia, nausea, vomiting, constipation, groin discoloration, dysuria, and a history of failed primary hernia repair during childhood. The patient underwent emergent surgery to excise ischemic-necrotic portions of the sigmoid colon, creation of end-colostomy, non-mesh repair of inguinal hernia, and right-sided complete orchiectomy with the removal of adjacent scrotal-Dartos tissues and spermatic cord due to Fournier’s Gangrene. This report provides both a report for a potentially preventable consequence in one of the most common surgical presentations and a review of the multi-disciplinary expertise that is required in the surgical management of complex inguinal hernias.

## Introduction

Inguinal hernias are among the most common acute abdominal diseases that present in the Emergency Department (ED) [1,2]. Classically, inguinal hernias involve the displacement of abdominal, pelvic, or groin tissue through a weakness in the abdominal wall, direct inguinal hernia [3]. The majority of direct inguinal hernias are uncomplicated and managed either conservatively or with routine/elective minimally invasive surgery [1,2]. However, under rare circumstances, inguinal hernias are susceptible to significant complications requiring emergent surgery [1,4]. Known complications can include hernia sac incarceration and strangulation, with increased risk in patients with obesity and concomitant disease contributing to increased intraabdominal pressure [4,5]. Furthermore, in the setting of a complicated inguinal hernia, there is a risk of vascular compromise to surrounding tissue as a product of the mass effect from the herniated tissue [4,6]. In the setting of complicated inguinal hernias with subsequent vascular, emergent multi-disciplinary surgical management is required: repair the originating hernia, manage abdominal complications, excise ischemic tissue to minimize risk for consequent necrotic infection, and potentially revascularize possible salvageable tissue [3-6].

Complicated strangulated inguinal hernias present acutely with non-specific symptoms indicative of abdominal obstruction: lower abdominal pain, nausea and vomiting, erythematous and tender areas of the overlying skin, and possible signs of septic shock [5,6]. However, additional clinical signs should be elicited to evaluate for secondary complications. This involves evaluating the surrounding anatomical regions for possible secondary ischemia, herniation, or perforation [5-9].

In this report, we review the case of a complicated strangulated inguinal hernia of the sigmoid colon with consequent vascular compromise of the ipsilateral scrotum and dartos tissue. This case required an emergent multi-disciplinary exploratory laparotomy to repair the hernia, debridement of necrotic tissue in the abdomen and scrotum, excision of necrotic tissue within the colon and scrotum, and revascularization with preservation of salvageable tissue.

## Case presentation

This is the case of a 61-year-old Hispanic-American male with a past medical history of obesity and recurrent inguinal hernia. Previous surgical history included a pediatric repair of an inguinal hernia at age eight, without mesh. The patient presented to the ED with a one-week history of lower-right abdominal pain that worsened in the past three days, prompting him to seek treatment. The patient also reported a three-day history of nausea, two episodes of vomiting, two days of constipation, a one-day discoloration of the groin, and a one-day history of dysuria.

**Figure 1:**
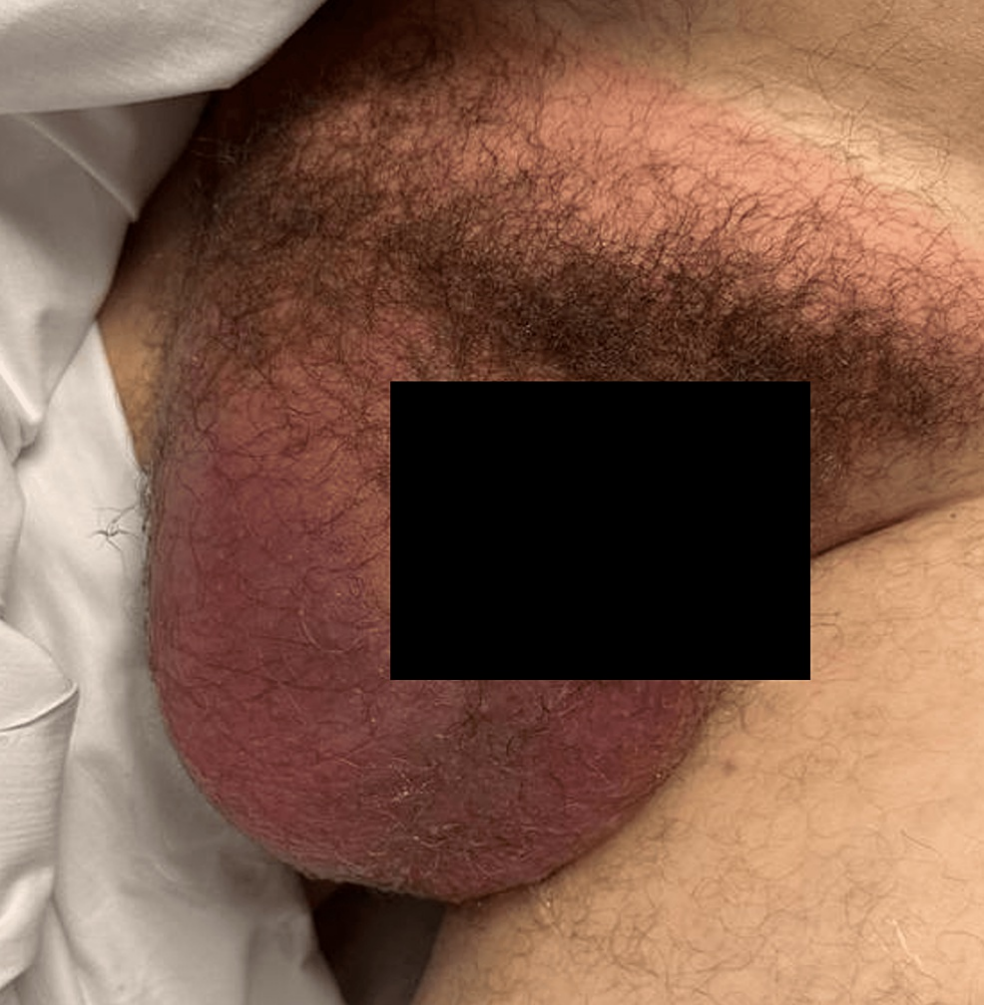
Erythematous lower-right inguinal hernia The hernia was non-reducible and tender on palpation; an extension to the groin with overlying warm-erythematous skin of scrotal tissue.

On presentation to the ED, the patient was in generalized discomfort, feverish (100.5°F/38.06°C), hypertensive (133/78mmHg), and tachycardic (104 beats per minute). Examination of the abdomen revealed an erythematous lower-right abdominal hernia, measuring 15x10cm, extending to the right scrotum (Figure 1). On physical examination, the hernia was firm, non-reducible, and severely tender to palpation. Further physical examination of the groin demonstrated a warm, erythematous scrotum, tender to palpation, and negative cremasteric reflex.

Complete Blood Count (CBC) identified an elevated White Blood Cell (WBC) count at 23,500/ul with neutrophilic shift, 93.7%. Inflammatory markers were also elevated; Erythrocyte Sedimentation Rate (ESR) and C-Reactive Protein (CRP) were 68.0 mm/hr and >350 mg/L respectively. The serum sodium level was 135 mEq/L, with an estimated Laboratory Risk Indicator for Necrotizing Fasciitis (LRINEC) score of 6. Additional preliminary blood tests revealed an elevated Lactic Acid, 2.6 mmol/L. This composition of patient history, clinical symptoms, and laboratory results were indicative of strangulated inguinal hernia with possible ischemic extension to the right scrotum. The patient was referred for intravenous (IV) contrast-enhanced CT-imaging of the abdomen and pelvis, ultrasound of the right scrotum, and urology consult due to concerns for ischemic-necrosis consequent to a strangulated inguinal hernia with possible ischemia of the right scrotum.

**Figure 2:**
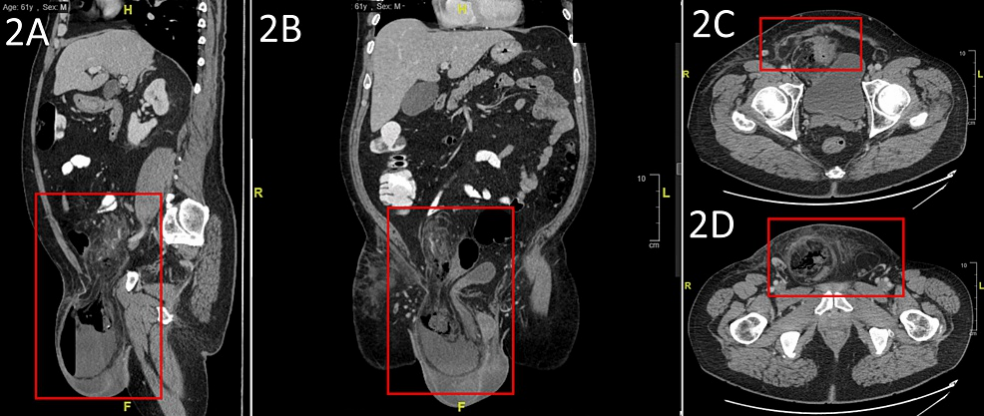
CT-imaging of a large, indurated right-sided inguinal hernia (A) Multiple loops of small bowel in the hernia-sac, possibly sigmoid colon; (B) Imaging also demonstrates possible herniation of right scrotal tissue; (C & D) Significant fat-stranding of adjacent soft tissues.

CT imaging (Figure 2) revealed an indurated right-sided inguinal hernia with multiple loops of small bowel and sigmoid colon within the sac (Figure 2A). Imaging suggested possible devolvement of the right scrotum into the hernia sac (Figure 2B). Furthermore, there was significant fat-stranding of the adjacent soft tissues (Figure 2C).

**Figure 3:**
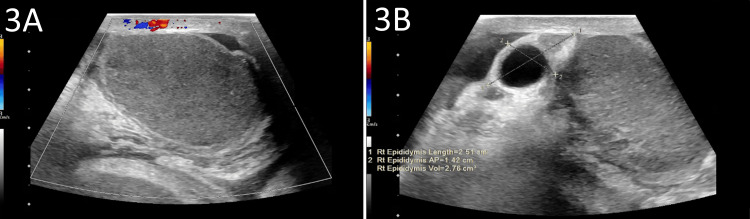
Ultrasound images of scrotum (A) The image demonstrates absent blood flow to the enlarged right testicle, measuring 4.2 x 2.8 x 2. 8 cm; (B) Ultrasound also reveals an enlarged right epididymis, measuring 2.5 x 1.4 cm.

Ultrasound (Figure 3) of the right scrotum demonstrated absent blood flow to an enlarged right testicle (Figure 3A), developing right-sided complex hydrocele, and enlarged right epididymis (Figure 3B). These radiological findings concerned ischemic necrosis of the strangulated herniated bowel loops and possible ischemia to the right testicle. The patient was referred for emergent exploratory laparotomy for repair of right-sided strangulated inguinal hernia, resection of ischemic bowel, creation of end-colostomy, and debridement of the right testicle.

**Figure 4:**
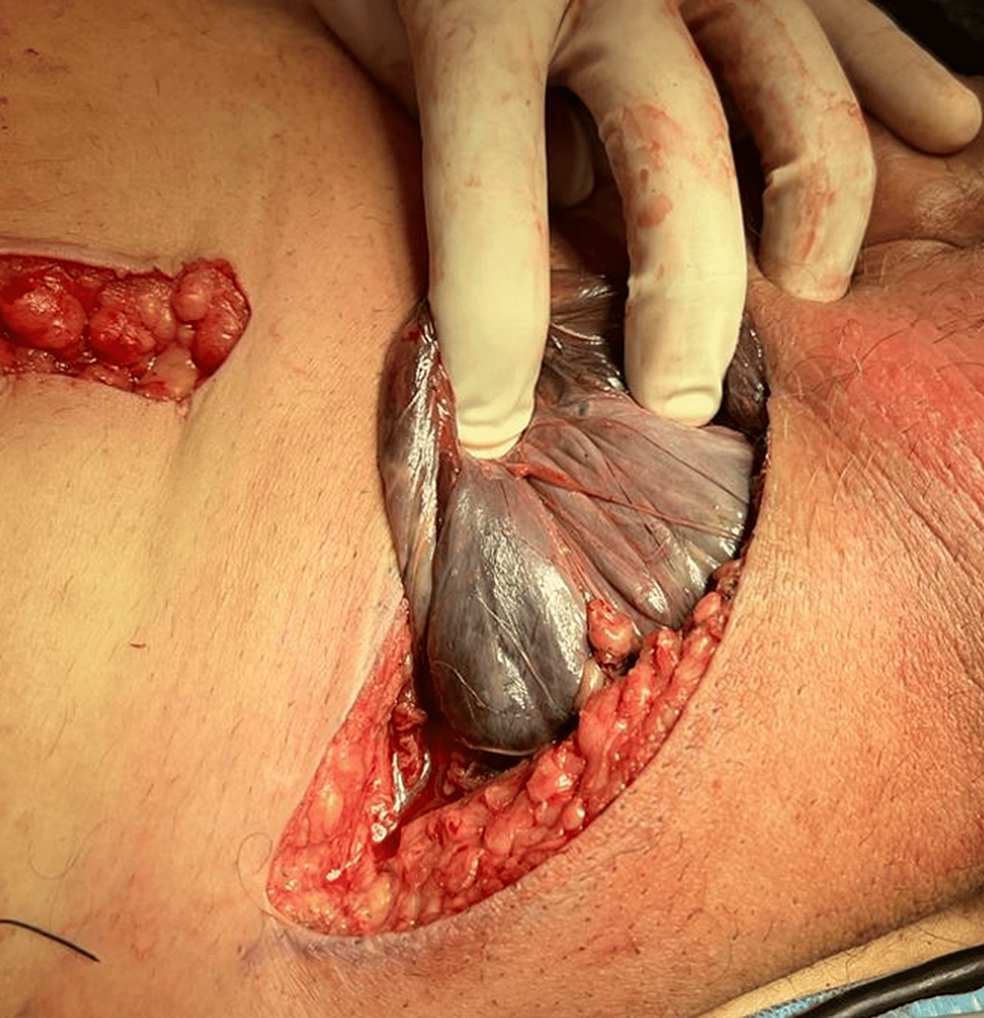
Intraoperative image 10 cm of sigmoid colon that was herniated into a sac was found to be ischemic, partially necrotic, and non-viable; transacted and converted into end-colostomy.

The patient underwent emergent exploratory laparotomy; during the inspection, a right-sided inguinal hernia containing a sigmoid colon was visualized. Utilizing a bowel grasper, the herniated sigmoid colon was partially reduced, revealing significant ischemic portions and copious amounts of feculent murky free fluid. On examination, 10 cm of sigmoid colon was found to be ischemic and non-viable (Figure 4), with the remainder of the herniated bowel demonstrating no abnormalities. The ischemic, partially necrotic, sigmoid colon was transected with a distal point identified for the creation of the end colostomy. Afterward, inguinal hernia repair was performed. During repair, gross infection of the scrotal tissue, ischemia of the spermatic cord, and necrosis of the right testicle and hernia sac were found. Full right-sided orchiectomy and debridement were performed. Primary repair of the hernia was performed due to concerns for a contaminated surgical field and increased risk for post-operative infection of the mesh.

The patient was discharged on postoperative day 10, following an uneventful hospital course, and transferred to short-term rehabilitation with pelvic drain, temporizing end-colostomy, and negative-pressure wound vacuum-assisted closure (VAC) on the open surgical site of the right groin. Upon discharge, the patient was afebrile, with a normalized WBC count of 9,500/ul, negative wound culture, and steady vancomycin-trough levels. The patient followed up with the outpatient clinic on postoperative day 18 for removal of pelvic drain, switch of negative-pressure wound VAC to regular sterile-dressing changes, switch to topical bacitracin, and planning for six-month ostomy-reversal. Outpatient appointment with urology for closure of open surgical wound in right groin occurred on postoperative day 21 with no further complications noted to date.

## Discussion

The lifetime prevalence of groin hernia is estimated between 27-43% in males, with inguinal hernia being the most common [1-3]. Nationally, primary inguinal hernia repair remains the most common abdominal procedure, estimated annually to be over 500,000 [2,3]. The most common indication for surgical repair of inguinal hernia is either elective for cosmetic repair or persistent-recurrent symptomatic hernias [1,3,4]. Recurrent inguinal hernias are susceptible to significant complications, including incarceration, strangulation, and vascular compromise of surrounding tissues, all of which require emergent surgical repair [1,4-6]. Therefore, preventative surgical repair has been suggested for patients with recurrent symptomatic inguinal hernias [3-6]. These comorbid risk factors include obesity, female sex, multiparity in females, chronic cough, chronic constipation, and previous abdominal surgery [1,3-7]. Complicated inguinal hernias warrant comprehensive assessment, often requiring multi-disciplinary surgical intervention to reduce and repair the instigating herniation, manage consequent abdominal complications, address ischemic and necrotic tissue, and potentially revascularize salvageable tissue [3-9]. Lastly, consideration should be given to the surgical repair of sliding inguinal hernias, with an overall incidence of 6-8% in inguinal hernias. In the setting of a sliding inguinal hernia, where the hernia sac is composed of visceral-retroperitoneal organs covered by the surrounding mesentery, increased surgical awareness must be given to prevent damage to the internal organ components of the hernia sac [3,10]. Surgical repair is then focused on peritonealization of the retroperitoneal contents of the sliding hernia sac with the extraperitoneal components utilized for repair of the defect [3-6,10]. As seen in this case, the patient required multidisciplinary surgical management to address both abdominal and urological complications in this case of strangulated inguinal hernia with ischemic orchitis of the right testicle and consequent Fournier gangrene. Consideration should be given to whether the patient would have benefited from early surgical intervention of persistent inguinal hernia, albeit asymptomatic, in the setting of contributing chronic comorbidities and failed primary pediatric repair.

Characteristically, an inguinal hernia presents with a reducible unilateral lower-abdominal/pelvic mass [4]. The diagnosis of simple inguinal hernia is primarily clinical and requires no additional diagnostic study beyond assessment on physical exam [1,4]. However, when the abdominal mass is irreducible, a diagnosis of hernia-incarceration is determined, requiring a more comprehensive diagnostic evaluation, laboratory blood tests, diagnostic imaging, and additional subspecialty consultation to evaluate for secondary consequences [4,11]. However, with hernia incarceration, clinical awareness of hernia strangulation and associated complications should be heightened [4]. Furthermore, complex strangulated inguinal hernias present acutely with non-specific abdominal symptoms: lower abdominal pain, nausea and vomiting, warm and erythematous skin, and presenting indications of fever and sepsis [5-7]. Given the nature of complex inguinal hernia, additional clinical signs should be obtained to assess for secondary non-abdominal complications due to secondary vascular compromise [5-9]. Complex strangulated inguinal hernia often requires emergent surgical intervention with multidisciplinary surgical support to correct these associated complications [7,9,11]. 

As reflected in this case, an obese male with failed primary hernia repair during childhood and longstanding recurrent inguinal hernia presented with a complex strangulated hernia associated with multiple associated complications: ischemia of a portion of the distal sigmoid colon, necrosis of the abdominal wall hernia sac and adjacent omentum, and right-sided testicular infarction resulting in complete orchiectomy of the right testicle. Albeit asymptomatic, the clinical circumstances surrounding this case raise the question of whether early-preventative surgical intervention in longstanding recurrent inguinal hernias may provide a favorable risk-benefit profile in certain patients. With the advancement and resource-optimization of minimally invasive abdominal surgery, the question of surgical repair vs watchful waiting may require re-evaluation [4,12]. The current guidelines suggest that watchful waiting is the preferred route in treatment for asymptomatic non-incarcerated inguinal hernia, opting for surgical repair only in elective settings [4,13,14]. However, the guidelines do not make adequate adjustments for comorbid associations that may increase the risk for hernia-incarceration, hernia-strangulation, or complex hernia consequences [12-14].

Systematic reviews of asymptomatic inguinal hernia estimated an average of 79% (range 48-91%) of patient-crossover rate from watchful waiting to surgical repair for worsening of hernia symptoms, 24% experiencing complex hernia complications, including ischemia, necrosis, septic shock, bowel perforation, and urinary complications [15,16]. This suggests that watchful waiting, while an effective temporizing measure in asymptomatic inguinal hernias, the majority still eventually require surgical repair with a significant proportion experiencing complex secondary complications with the potential for disastrous consequences [12,15,16]. Therefore, for patients with significant contributing comorbidities that increase the risk for complex hernia development, similar to those of our patient, early-preventative minimally invasive surgical management may provide a clinically favorable treatment strategy that curtails the development of disastrous consequences [13]. However, significant, comprehensive, and meticulous investigation is required to provide evidence to this hypothesis.

## Conclusions

Inguinal hernias are among the most prevalent acute abdominal diseases. Surgical intervention is usually reserved for symptomatic, complicated, or complex hernias. Asymptomatic inguinal hernias are usually managed with watchful waiting, without adequate consideration of contributing comorbidities that would increase the risk for progressively worsening disease or hernia-associated complications. With the rapid evolution, optimization, and popularization of minimally invasive surgical repair, preventative early surgical intervention may provide a favorable clinical course and reduce the development of significant clinical consequences. This report provides both a report for a potentially preventable consequence in one of the most common surgical presentations and a review of the multi-disciplinary expertise required in the surgical management of complex inguinal hernias.
